# Adh Promotes *Actinobacillus pleuropneumoniae* Survival in Porcine Alveolar Macrophages by Inhibiting CHAC2-Mediated Respiratory Burst and Inflammatory Cytokine Expression

**DOI:** 10.3390/cells12050696

**Published:** 2023-02-22

**Authors:** Junhui Zhu, Rining Zhu, Hexiang Jiang, Ziheng Li, Xuan Jiang, Fengyang Li, Fuxian Zhang, Xin Feng, Jingmin Gu, Na Li, Liancheng Lei

**Affiliations:** 1 College of Veterinary Medicine, Jilin University, Changchun 130062, China; 2 College of Animal Science, Yangtze University, Jingzhou 434025, China

**Keywords:** *Actinobacillus pleuropneumoniae*, Adh, CHAC2, immune invasion, survival

## Abstract

*Actinobacillus pleuropneumoniae* (*A. pleuropneumoniae*) causes porcine pleuropneumonia that seriously endangers pig’s health. Adh, located in the head region of trimeric autotransporter adhesion of *A. pleuropneumoniae*, affects bacterial adhesion and pathogenicity. However, how Adh mediates *A. pleuropneumoniae* immune invasion is still unclear. Here, we established the *A. pleuropneumoniae* strain L20 or L20 ΔAdh-infected porcine alveolar macrophages (PAM) model, and applied protein overexpression, RNA interference, qRT-PCR, Western blot and immunoflourescence techniques to dissect the effects of Adh on PAM during *A. pleuropneumoniae* infection. We found that Adh could increase the *A. pleuropneumoniae* adhesion and intracellular survival in PAM. Gene chip analysis of piglet lungs further showed that Adh significantly induced cation transport regulatory-like protein 2 (CHAC2) expression, whose overexpression suppressed the phagocytic capacity of PAM. Furthermore, CHAC2 overexpression dramatically increased glutathione (GSH) expression, decreased reactive oxygen species (ROS), and promoted *A. pleuropneumoniae* survival in PAM, while the knockdown of CHAC2 reversed these phenomena. Meanwhile, CHAC2 silence activated the NOD1/NF-κB pathway, resulting in an increase in IL-1β, IL-6, and TNF-α expression, whereas this effect was weakened by CHAC2 overexpression and addition of NOD1/NF-κB inhibitor ML130. Moreover, Adh enhanced the secretion of LPS of *A. pleuropneumoniae*, which regulated the expression of CHAC2 via TLR4. In conclusion, through a LPS-TLR4-CHAC2 pathway, Adh inhibits respiratory burst and inflammatory cytokines expression to promote *A. pleuropneumoniae* survival in PAM. This finding may provide a novel target for the prevention and treatment of *A. pleuropneumoniae*.

## 1. Introduction

*Actinobacillus pleuropneumoniae* (*A. pleuropneumoniae*) is a highly infectious pathogen of swine respiratory tract disease that can cause severe lung damage and death [1]. The adaptability and persistence of *A. pleuropneumoniae* in the host are closely related to its infection, making it difficult for host immune system to clearance of this pathogen. Several virulence factors of *A. pleuropneumoniae* influence its pathogenicity [2]. For example, trimeric autotransporter adhesion (TAA), a newly discovered virulence factor, has aroused the industry’s interest [3,4]. Previous studies suggested that TAA of *A. pleuropneumoniae* was Apa1 with a critical functional area, the head region structure (Adh), the absence of which could significantly reduce the virulence and adhesion of *A. pleuropneumoniae* [3,5,6]. Moreover, *Adh* could attenuate the expression levels of antigen presentation genes and inhibit host immune response [3]. However, the mechanisms of *A. pleuropneumoniae* survival in host have not been fully elucidated. Further exploration on Adh’s role in regulating *A. pleuropneumoniae* immune escape is particularly important.

Macrophages are essential immune cells for bacterial infection and have various complex functions in bacteria elimination [7]. Additionally, particle uptake and phagosome maturation methods by macrophages may also contribute to bacteria clearance [7]. Therefore, bacterial intracellular survival depends on a multilevel tug of war between the pathogen and host macrophages. The survival of the pathogenic bacteria constantly improves their capability for host invasion, colonization, and evasion to immune system [7,8,9]. To evade the immunological clearance by macrophages, several bacteria have developed a spectrum of strategies, including interfering internalization, affecting phagocytic mechanisms, and reducing detoxifying enzymes levels [7,9]. To date, numerous studies have shown that many pathogens may evade the innate immune defense like *Klebsiella pneumoniae* [7,8,10].

*CHAC2* is a newly discovered gene with a contentious role in GSH degradation. Several studies have found the function of degrading GSH as *CHAC1* in cancer; however, the role of *CHAC2* in GSH degradation remains controversial in different cancers [11,12]. It is important to investigate the regulation of *CHAC2* on its homeostasis for host defense. *However,* the role of *CHAC2* in mediating *A. pleuropneumoniae* survival in macrophages and evading macrophage eradication mechanisms remain unexplored. *A. pleuropneumoniae* break the innate immune defense of macrophage, leading to persistent infection, and this effect was attenuated by *Adh* deletion [3]. However, the exact mechanism by which *Adh* mediates *A. pleuropneumoniae* immune invasion is still unknown. In this study, we found that *Adh* increases the *CHAC2* expression level in PAM through LPS-TLR4 pathway. Moreover, *CHAC2* inhibited the ROS and inflammatory cytokines expression, which led to *A. pleuropneumoniae* survival in PAM. Our results reveal the new evidence that *A. pleuropneumoniae* induces immune escape by *CHAC2* manipulating macrophage’s respiratory burst.

## 2. Materials and Methods

### 2.1. Bacteria Strains, Cells and Culture Conditions

The strains of *A. pleuropneumoniae* serotype 5b strain L20 and L20 *Adh* deletion (Δ*Adh*) were maintained and described in our laboratory previously [3]. All strains were grown in brain–heart infusion broth (BD Biosciences, San Jose, CA, USA) with 20 μg/mL of nicotinamide dinucleotide (Sigma-Aldrich, St. Louis, MO, USA) at 37 °C [3,13]. Immortalized PAM (ATCC, CRL-2845) was provided by the Harbin Veterinary Research Institute of the Chinese Academy of Agricultural Sciences, and cultured in DMEM (containing 100 U/mL of penicillin and 100 μg/mL of streptomycin) supplemented with 10% FBS (Clark Bioscience, Richmond, VA, USA) at 37 °C and 5% CO_2_ in an incubator.

### 2.2. Regents, Antibodies and Plasmids

The antibodies, reagents and plasmids used in this study were as follows: X-tremeGENE HP DNA transfection reagent (Roche Diagnostics, Grenzach, Germany); anti-CHAC2 (Proteintech, Rosemont, IL, USA); anti-p65 (Affinity Biosciences, Changzhou, China); anti-pp65 (Affinity Biosciences, Changzhou, China); anti-GAPDH (Cell Signaling Technology (CST), Danvers, MA, USA); anti-NOD1 and anti-TLR4 (Affinity Biosciences, Changzhou, China), ML130 inhibitor and TAK-242 (Selleck, Houston, TX, USA), pcDNA3.1 plasmids (Invitrogen, Carlsbad, CA, USA).

### 2.3. Adhesion and Intracellular Survival Assays

Adhesion and intracellular survival assays were conducted in PAM according to a previous study [14]. Each well of 6-well plates was seeded with 1 × 10^6^ PAM and grown at 37 °C, 5% CO_2_ for 12 h (h). *A. pleuropneumoniae* strain L20 or Δ*Adh* (MOI = 100) were co-cultured with PAM for 0.5 and 1 h, washed 3 times with PBS, respectively. Cells were then digested by trypsin to obtain bacteria, which was diluted into 10^3^~10^5^ dilution titers and plated on BHI solid medium. Three replicates per titer were counted next day.

The conditions for the intracellular survival assay were the same as described above for the adhesion assay. Briefly, the samples were rinsed 3 times with PBS at 0.5 h, 1 h, and 2 h. Sequentially, gentamicin (50 μg/mL final concentration) was added, incubated at 37 °C for 20 min, and washed 3 times. Finally, the PAM were lysed with 1 mL 0.1% Triton X-100 (Sigma-Aldrich, St. Louis, MO, USA) for 20 min at 37 °C, and the samples were centrifuged for 8 min at 8000× *g*. After obtaining the bacteria, similar procedures were performed as adhesion assay to count the number of bacteria.

### 2.4. Immunofluorescence

Immunofluorescence was performed as previously described [10]. Briefly, 1 × 10^5^ PAM were seeded into 12-well plates, infected with *A. pleuropneumoniae* strain L20 or Δ*Adh* at MOI of 10:1, and incubated at 37 °C for 2 h, respectively. The cells were washed 3 times with PBS, fixed by 4% paraformaldehyde for 30 min and penetrated with 0.5% Triton X-100 for 15 min. After 3 additional washes in PBS, cells were blocked with 10% goat serum for 1 h at room temperature and incubated overnight with the corresponding primary antibodies at 4 °C. Cells were rinsed 3 times in PBS and then incubated with blocking buffer containing Alexa-Fluor 488 secondary antibody for 50 min at room temperature. Imaging experiments were conducted using an inverted fluorescence microscope (Olympus, Tokyo, Japan) and analyzed with the Image-J software (National Institutes of Health, Bethesda, MD, USA).

### 2.5. RNA Extraction and qRT-PCR

For 2 h, PAM were co-cultured with *A. pleuropneumoniae* strain L20 or Δ*Adh* at MOI of 10:1, respectively. After that, the cell supernatant was obtained and detected with an IL-10 ELISA kit (Jinma Biotechnology, Shanghai, China). RNA was extracted using the total RNA Kit according to the manufacturer’s instructions (Bioer, Hangzhou, China), and reverse-transcribed into cDNA with a Prime Script RT reagent Kit (Takara, Dalian, China). The cDNA was then mixed with cytokine primers (Table S1) and TB Green Premix Ex Taq II (Takara, Dalian, China) used for qPCR of 20 reaction volumes, amplified cDNA on the Quant Studio 3 qPCR system (ABI Biosystems, Foster City CA, USA). Finally, the 2^−ΔΔCt^ method was used to calculate relative gene expression [15].

### 2.6. Gene Chip Was Used to Detect the Expression Profile of Lung Cells

DNA microarray technology was used to find lung gene expression signatures 48 h after *A. pleuropneumoniae* infection (n = 3, PBS control, L20 and Δ*Adh*) [3]. RNA was extracted to obtain cDNA to use Affymetrix gene chip sequencing by Qi Ming Bio (Qiming, Shanghai, China). Sequencing data analysis was used a random variance model to assess Adh-regulated genes. The animal study was reviewed and approved Animal Welfare and Research Ethics Committee of Jilin University (Number of permits:20121202).

### 2.7. Construction and Verification of the Plasmid pcDNA-CHAC2 and shRNA-CHAC2

The method was the same to 2.5 to obtain cDNA and build a 25 μL RT-PCR reaction system with primers (Table S1) and rTaq MIX (Takara, Dalian, China) to amplify the CHAC2 gene. PcDNA3.1 was digested with *Hind III* and *ECOR I* (Takara, Dalian, China) and purified using the SanPrep Column PCR Product Purification Kit (Sangon Biotech, Shanghai, China). Beyotime Biotechnology Company’s Seamless Cloning Kit (Beyotime, Shanghai, China) was used to assemble the pcDNA-CHAC2 plasmid using the 1:3 ratio of PCR fragments and plasmids in the connection system. The short hairpin RNA (shRNA) on CHAC2 (the sequence of CHAC2-A, B, and C (Table S2)) was synthesized by PPL (Public Protein/Plasmid Library, Nanjing, China). The pcDNA-CHAC2 plasmid and shRNA-CHAC2 (A, B, and C) were together to be tested and verified.

### 2.8. Western-Blot

PAM were stimulated for 2 h with *A. pleuropneumoniae* strain L20 or Δ*Adh*, then incubated on ice with RIPA lysis buffer (Solarbio, Beijing, China) and PMSF (130:1) to obtain protein. The protein was collected for BCA (Thermo, New York, NY, USA) assay analysis and western-blot assays. Briefly [5], the samples were subjected to SDS-PAGE and PVDF membrane transfer, in which 5% nonfat milk powder was blocked at room temperature for 2 h on a shaker. The primary antibodies CHAC2, NOD1, p65, p-p65, and TLR4 were incubated overnight on the membrane at 4 °C. They were then washed 3 times with PBST buffer and finally incubated with HRP-conjugated secondary antibody for 50 min at room temperature. The exposure imaging was carried out using ECL (Merck, Burlington, VT, USA), the quantitative signal analysis was performed using Image-J (National Institutes of Health, Bethesda, MD, USA).

### 2.9. Phagocytosis Experiments

Phagocytosis experiment were prepared as previously described with modifications [16]. The impact of *CHAC2* on the phagocytic capacity of PAM was investigated using fluorescent microspheres. When the PAM reached 70% confluence, they were washed once with PBS and then replaced with serum-free media, which were then passed once more. All steps of the pcDNA-CHAC2 and shRNA-CHAC2 plasmid transfection were carried out using the X-tremeGENE HP DNA kit (Roche Diagnostics, Grenzach, Germany) according to the manufacturer’s instructions. A fluorescent microsphere working solution was prepared by combining fluorescent microspheres (BaseLine, Tianjin, China) with 1% BSA in DMEM in an incubator at 37 °C, for 30 min in the dark. Then, the microsphere working solution was added to the transfected PAM cells, which were incubated for 3 h at 37 °C in the dark. Finally, PAM cells were washed 3 times with PBS and fluorescent signals in PAM cells were collected by fluorescence microscope (Rate of phagocytosis = phagocytosis of cells with fluorescent microspheres/the total number of cells in view).

### 2.10. GSH Detection

As described in 2.9, pcDNA-CHAC2 and shRNA-CHAC2 plus their vector control were transfected into PAM for 24 h, respectively, and then *A. pleuropneumoniae* strain L20 and Δ*Adh* strains were challenged with MOI of 10:1 for 2 h, respectively. The Beyotime total GSH detection kit (Beyotime, Shanghai, China) was used to detect GSH followed the manufacturer’s instructions. After that, the absorbance was caught by a microplate reader (Biotek, Winooski, VT, USA), and the content of GSH was calculated.

### 2.11. ROS Level Detection

After pcDNA-CHAC2 and shRNA-CHAC2 were transfected, ROS were detected using the Beyotime Biological ROS Detection Kit (Beyotime, Shanghai, China) and recorded using a fluorescence microscope. The fluorescence intensity of DCFH-DA was evaluated using Image-J software (National Institutes of Health, Bethesda, MD, USA).

### 2.12. LPS Detection

The *A. pleuropneumoniae* infection method refers to previous Section 2.5. LPS was measured by Endotoxin Detection System (GenScript, Nanjing, China). All procedures refer to the method provided by the Toxin Sensor Chromogenic LAL Endotoxin Assay Kit. Briefly, we added limulus amebocyte lysate (LAL) reagent to the standards and samples and incubated them at 37 °C. Then, chromogenic substrate was incubated for 6 min at 37 °C. The stop solution halted the reaction. The final step is to read the absorbance at 545 nm by microplate reader (BioTek, Winooski, VT, USA) and use the standard as a reference to determine the sample’s composition.

### 2.13. Statistical Analysis

GraphPad Prism 8.3 (GraphPad Prism Software Inc., San Diego, USA) was used for all statistical analyses. All data are shown as means ± standard deviation. One-way analysis of variance (ANOVA) and *t*-test were used to determine the statistical differences. NS *p* > 0.05, * *p* < 0.05, ** *p* < 0.01, and *** *p* < 0.001.

## 3. Results

### 3.1. Adh Deletion Decreases A. pleuropneumoniae Adhesion, Intracellular Survival in PAM and Cytokine Expressions

**Figure 1:**
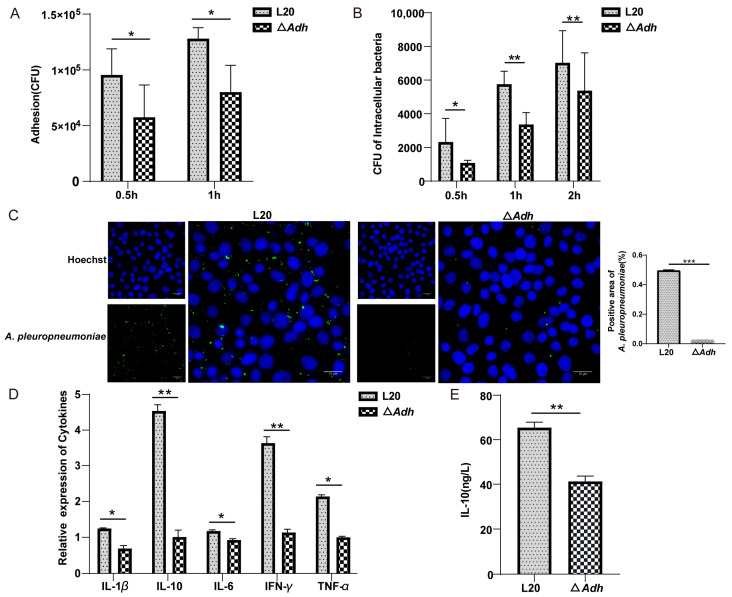
Adhesion ability, intracellular bacterial count of *A. pleuropneumoniae* strain L20, Adh deletion mutant (Δ*Adh*), and cytokine expressions in PAM. (**A**) Adhesion assay. (**B**,**C**) The number of intracellular bacteria were counted using the plate method (**B**) and immunofluorescence analysis (**C**). (**D**) Expression of inflammation-associated cytokines in PAM infected with L20 or Δ*Adh*. (**E**) IL-10 secretion of PAM was detected by ELISA. Multiple *t*-test and *t*-test. * *p* < 0.05, ** *p* < 0.01, ***, *p* < 0.001.

PAM, as a key immune subset in the lung, is critical in elimination of germs during bacterial infection. The *Adh* deletion mutant (Δ*Adh*) and *A. pleuropneumoniae* strain L20 were used to infect PAM to compare the *A. pleuropneumoniae* adhesion and intracellular survival. We found that the Δ*Adh* strain decreased bacterial adhesion capacity compared to the *A. pleuropneumoniae* strain L20 at both 0.5 h and 1 h (*p* < 0.05) (Figure 1A). Furthermore, the number of intracellular bacteria of the Δ*Adh* strain was also shown to be significantly lower than that of the *A. pleuropneumoniae* strain L20 at 0.5 h, 1 h, and 2 h (Figure 1B). These results were also verified by immunofluorescence assays (Figure 1C). Meanwhile, the qPCR results showed that *IL-1β*, *IL-6, TNF-α, IFN-γ*, and *IL-10* expression of PAM were also significantly reduced in the Δ*Adh* group than *A. pleuropneumoniae* strain L20 (Figure 1D). The contents of IL-10 were also confirmed by ELISA (Figure 1E). Taken together, these results indicated that *Adh* deletion reduces *A. pleuropneumoniae* adhesion to PAM, intracellular bacterial survival, and cytokine expressions.

### 3.2. CHAC2 Expression Inhibits the Phagocytic Capacity of PAM

**Figure 2:**
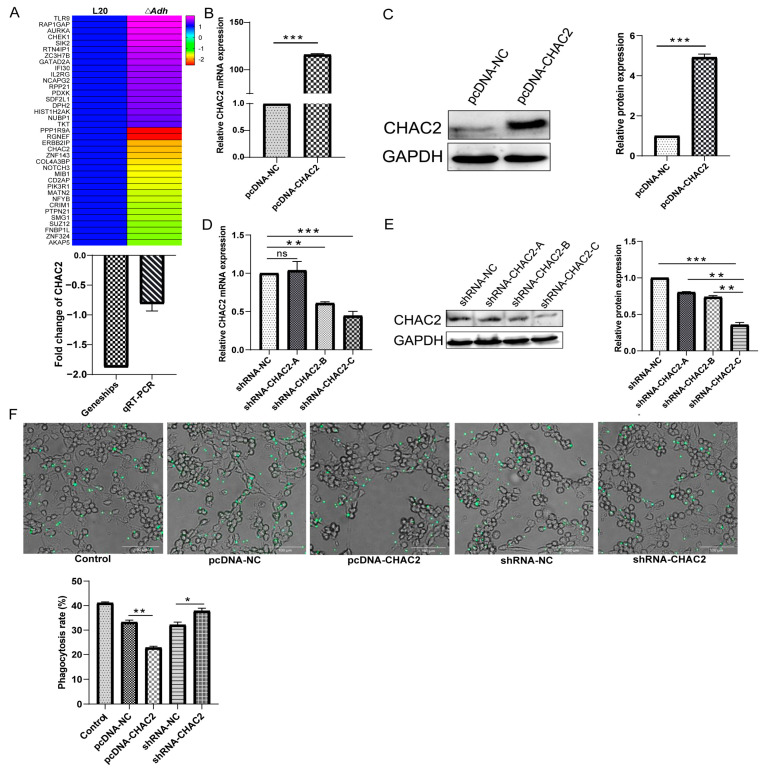
CHAC2 overexpression plasmid and shRNA plasmid were constructed to analyze the phagocytic capacity of PAM. (**A**) Heatmap of differential gene expression profiles in piglet lungs induced by *Adh*. (**B**,**C**) Detection of (**B**) mRNA and (**C**) protein expression level of pcDNA-CHAC2. (**D**,**E**) Detection of (**D**) mRNA and (**E**) protein expression level of shRNA-CHAC2. (**F**) Phagocytic fluorescent microspheres were used to evaluate the impact of CHAC2 on PAM phagocytosis. *t*-test and One-way ANOVA. NS *p* > 0.05, * *p* < 0.05, ** *p* < 0.01, and *** *p* < 0.001.

In order to dissect the above mechanism of *A. pleuropneumoniae* immune escape mediated by *Adh*, gene chip was performed on the piglet lung tissue infected with *A. pleuropneumoniae* strain L20 and Δ*Adh*, respectively. This analysis yields a total of 495 differential genes between these two groups. After removing the same genes in the *A. pleuropneumoniae* strain L20 and the control group, we obtained 37 differential gene expressions that were regulated in the lung tissue (18 upregulated and 19 downregulated) (Figure 2A). Interestingly, *CHAC2*, a crucial regulator of cellular oxidative stress, was significantly downregulated in Δ*Adh* group, which was also validated by qRT-PCR (Figure 2A).

To further understand the function of *CHAC2* in the regulation of *A. pleuropneumoniae* immune evasion, an overexpression plasmid and shRNA of *CHAC2* was constructed (Figure S1A–C). The results of validation assay indicated that CHAC2 protein and mRNA expression levels significantly increased in pcDNA-CHAC2 group (Figure 2B,C). The quantitative analysis by Image-J and mRNA levels showed that shRNA-CHAC2-C mediated more effectively on *CHAC2* knockdown than shRNA-CHAC2-A/B (Figure 2D,E). Hence, shRNA-CHAC2-C was used as the major test item in the following assays. Next, fluorescent microsphere phagocytosis assay was performed to assess the phagocytic ability of PAM by CHAC2 regulation. The phagocytosis rate was significantly higher in pcDNA-NC compared with pcDNA-CHAC2 group. Similar, shRNA-CHAC2 group showed a significantly higher phagocytosis rate than shRNA-NC group (Figure 2F). Thus, these results suggested that *CHAC2* expression could suppress the phagocytic capacity of PAM.

### 3.3. CHAC2 Inhibits ROS Generation in PAM to Improve A. pleuropneumoniae Survival

**Figure 3:**
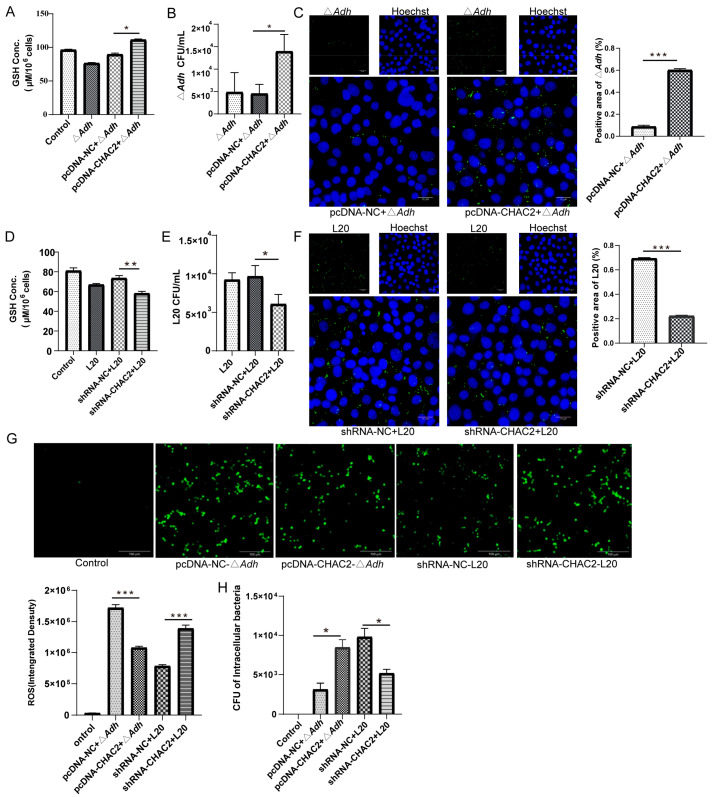
CHAC2 overexpression or suppression altered GSH, ROS, and intracellular bacterial counts during *A. pleuropneumoniae* infection. (**A**,**B**) Effects of treatment with pcDNA-CHAC2 on (**A**) GSH levels and (**B**) intracellular bacterial counts in PAM treated with Δ*Adh*. (**C**) Intracellular bacterial counts in Δ*Adh* by Immunofluorescence. (**D**,**E**) Effects of treatment with shRNA-CHAC2 on (**D**) GSH levels and (**E**) intracellular bacterial counts in PAM treated with *A. pleuropneumoniae* strain L20. (**F**) Intracellular bacterial counts in *A. pleuropneumoniae* strain L20 by Immunofluorescence. (**G**,**H**) Effects of treatment with *CHAC2* overexpression or inhibitor on (**G**) ROS levels (ImageJ analysis) and (**H**) intracellular bacterial counts in PAM treated with *A. pleuropneumoniae* (Δ*Adh* or L20). *t*-test and One-way ANOVA. * *p* < 0.05, ** *p* < 0.01, and *** *p* < 0.001.

*CHAC2* is an enzyme that regulates the GSH homeostasis in the cytosol of mammalian cells [17]. We further investigated whether *CHAC2* affect *A. pleuropneumoniae* strain L20 adhesion and the number of intracellular bacteria in PAM by modulating GSH levels. PAM were transfected with either pcDNA-CHAC2 or shRNA-CHAC2 and infected Δ*Adh* and *A. pleuropneumoniae* strain L20, respectively. GSH content and intracellular bacteria detection results showed that *CHAC2* overexpression could significantly increase GSH levels and the number of Δ*Adh* in PAM (*p* < 0.01) (Figure 3A–C). Similarly, we found that *CHAC2* knockdown reduced GSH levels and the amounts of *A. pleuropneumoniae* strain L20 in PAM (Figure 3D–F). As GSH is a non-protein thiol that scavenges ROS [18], we detected the ROS production after transfecting pcDNA-CHAC2 or shRNA-CHAC2 into PAM infected with Δ*Adh* or *A. pleuropneumoniae* strain L20. The results showed that overexpression of *CHAC2* reduced ROS levels compared to controls (*p* < 0.001) and protected Δ*Adh* from ROS-mediated killing (Figure 3G,H). Furthermore, shRNA-mediated *CHAC2* knockdown increased the ROS production and reduced *A. pleuropneumoniae* strain L20 intracellular survival in PAM (Figure 3G,H). Thus, these results provided the evidence that *CHAC2* overexpression can lead to the increase in cellular GSH to inhibit ROS production, and consequently facilitate *A. pleuropneumoniae* survival in PAM.

### 3.4. CHAC2 Inhibits Inflammatory Cytokine Expression by NOD1/NF-κB Signaling Path Way during A. pleuropneumoniae Infection

**Figure 4:**
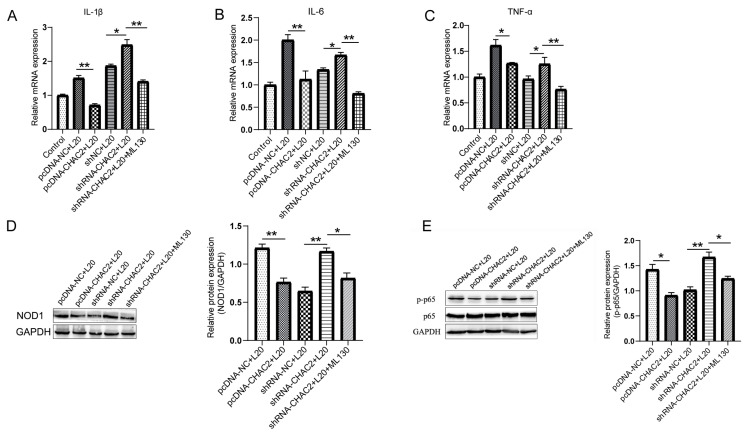
CHAC2 overexpression or suppression altered NOD1/NF-κB signaling pathway and cytokines expression during *A. pleuropneumoniae* infection. (**A**–**C**) mRNA expression levels of the inflammatory cytokines (**A**) IL-1β, (**B**) IL-6, and (**C**) TNF-α by CHAC2 regulation in *A. pleuropneumoniae* infection. (**D**,**E**) The levels of (**D**) NOD1 and (**E**) p-p65 were tested by Western blot analysis. Quantification of Western blot band intensities was performed using ImageJ. One-way ANOVA. * *p* < 0.05, ** *p* < 0.01.

To dissect the function of *CHAC2* in inflammation during *A. pleuropneumoniae* infection, PAM transfected with or without *CHAC2* knockdown and overexpression vectors were exposed to *A. pleuropneumoniae* strain L20 to measure the expression levels of inflammatory cytokines. The qRT-PCR cytokine detection results showed that silencing of CHAC2 aggravated the *A. pleuropneumoniae*-induced expression of proinflammatory factors *IL-1β*, *IL-6*, and *TNF-α*, while overexpression of CHAC2 blunted these cytokines expression (Figure 4A–C). Meanwhile, Western blot results showed that *CHAC2* knockdown significantly increased the expression of activated NOD1 and NF-κB (Figure 4D,E). In addition, the presence of the selective NOD1/NF-κB inhibitor ML130 abolished the proinflammatory effect of *CHAC2* deletion during *A. pleuropneumoniae* treatment (Figure 4A–E). These results indicated the regulatory role of *CHAC2* in inflammation during *A. pleuropneumoniae* infection was mediated by the NOD1/NF-κB signaling pathway.

### 3.5. CHAC2 Is Regulated by Adh via the LPS-TLR4 Pathway

**Figure 5:**
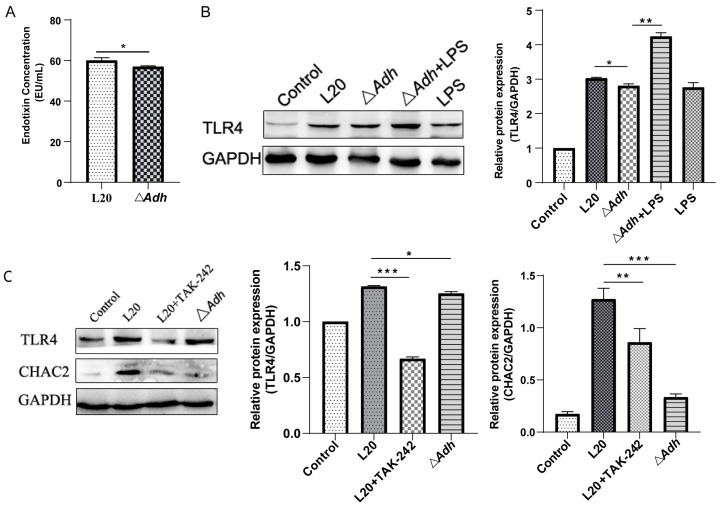
The effect of *A. pleuropneumoniae*’s LPS on the expression of TLR4 and CHAC2. (**A**) LPS detection in *A. pleuropneumoniae* strain L20 and Δ*Adh*. (**B**,**C**) The levels of (**B**) TLR4 and (**C**) CHAC2 were measured by Western blot analysis and ImageJ analysis. *t*-test and One-way ANOVA. * *p* < 0.05, ** *p* < 0.01, *** *p* < 0.001.

TLR4 have been shown to be vital for immune recognition of *A. pleuropneumoniae* [19], hence we examined the effects of *Adh* deletion on TLR4 expression and LPS. The results of the LPS assay showed that *A. pleuropneumoniae* strain L20 induced significantly higher LPS levels than Δ*Adh* (*p* < 0.05) (Figure 5A). Western blot analysis showed that *A. pleuropneumoniae* strain L20 induced significantly increased expression of TLR4 in PAM than Δ*Adh*, and the addition of LPS to Δ*Adh* increase the expression of TLR4 (Figure 5B). In line with the results of Figure 2A, the protein expression levels of *CHAC2* were also significantly increased in *A. pleuropneumoniae* strain L20 than Δ*Adh*, while TLR4 inhibitor TAK-242 impaired the *CHAC2* expression (Figure 5C). Therefore, these findings suggested that *Adh* regulated the expression of *CHAC2* via the LPS-TLR4 pathway.

## 4. Discussion

*A. pleuropneumoniae* is one of the most harmful pathogens for respiratory infections of pigs; whether acute or chronic, it frequently causes increased mortality of ill pigs [1]. *A. pleuropneumoniae* may be present in tonsillar crypts and chronic lung lesions in pigs that survive chronic or acute *A. pleuropneumoniae* infection [20]. In the current study, we found that *Adh* enhances the adhesion and intracellular survival of *A. pleuropneumoniae* in PAM. *Adh* increases the expression of *CHAC2* through LPS-TLR4 pathway, which reduces the bactericidal impact of ROS in PAM by the increase the amount of GSH and inhibits the pro-inflammatory cytokine expression through NOD1/NF-κB signaling pathway. For the first time, our results clarify the mechanism that *Adh* assist the *A. pleuropneumoniae* to escape from the host immune response.

Virulence factors play a vital role during *A. pleuropneumoniae* colonization and infection [21]. The *FTPA* gene of *A. pleuropneumoniae* has been identified as a unique DPS-like protein to boost the resistance of *A. pleuropneumoniae* to H_2_O_2_, promotes *A. pleuropneumoniae* survival in macrophages, and exacerbates the infection process in mice [22]. In our study, we found that *Adh* can regulate *CHAC2* to resist the oxidative killing by macrophages, which adds another functional gene to protect it from oxidative injury. Combined with the finding that *A. pleuropneumoniae* infection leads to the death of alveolar macrophages via toxins [23], our data suggested that the *A. pleuropneumoniae* may first increase the CHAC2 expression of macrophages to survive and proliferate in macrophages, and eventually kill macrophages. *A. pleuropneumoniae* has evolved comprehensive antioxidant mechanisms to evade immune surveillance and promote intracellular survival [22]. At present, research on *CHAC2* is mainly focused on human diseases, especially cancers. However, compared with another member, *CHAC1*, the functions of *CHAC2* remain poorly studied [11,12]. To our knowledge, we are the first to dissect the roles of this novel antioxidative target during bacterial infection in pigs. A recent study revealed that *CHAC2* was distinct from *CHAC1* in both sequence and function, of which *CHAC2* shows about 50% sequence identity and 10~20-fold lower catalytic efficiency than *CHAC1* [17]. Previous studies have shown that *CHAC2* tends to maintain rather than efficiently degrade GSH compared to *CHAC1* [17]. In addition, *CHAC2* can also dose-dependently suppress the degraded GSH by *CHAC1*. Kaur et al. discovered that *CHAC2* is not an efficient degradation of GSH but an enzyme to slow turnover of cytosolic glutathione [17]. GSH in the host is a ROS scavenger produced by γ-glutamyl cycle of glutathione acting to inhibit intracellular ROS [18]. We found that *CHAC2* could prevent ROS formation during *A. pleuropneumoniae* infection and allowed the *A. pleuropneumoniae* to suppress the macrophage-killing effect of ROS. This further enriches the immune invasion strategies of *A. pleuropneumoniae*.

The inflammatory response is critical for macrophage defense against invasive pathogens, such as immunity to *M. tuberculosis*, which is in part attributable to the activation of the inflammasome, a multiprotein complex that facilitates the killing of intracellular bacteria [8,24]. Interestingly, *M. tuberculosis* has also evolved to counteract the inflammatory response by secreting ZmpA that inhibits *IL-1β* processing by the host cells [8,24,25]. In this study, we found that CHAC2 knockdown can activate NOD1/NF-κB pathway, which leads to the production of inflammatory factors and an increase in the bactericidal effect of PAM. However, *A. pleuropneumoniae* infection significantly increased IL-10 production and reshaped the immune environment, making *A. pleuropneumoniae* more conducive to survive in PAM. *A. pleuropneumoniae* infection often generates a significant increase in the inflammatory cytokines *IL-1β*, *TNF-α*, and *IL-10* at 6 h, which recovers to normal levels between 12 and 24 h [5,26]. Previous research has demonstrated that *Adh* may trigger PAM apoptosis, and the death of PAM has a significant impact on the process of inflammatory cytokines release [5]. In this study, we discovered that the absence of *Adh* boosts the production of inflammatory cytokines via *CHAC2*, indicating that *Adh* may be a critical role in regulating inflammatory responses. LPS is a considerable glycolipid in the outer membrane of Gram-negative bacteria and can induce TLR4 expression to provoke an uncontrolled inflammatory response in macrophages [19,27]. Previous studies had shown that LPS synthesis genes were up-regulated in PAM during L20 infection [28]. In addition, the inactivation of LPS biosynthesis genes in *E. coli* has been linked to oxidative stress [29]. We found that *Adh* increased the expression of TLR4 by enhancing the secretion of LPS. However, the mechanism of Adh-mediated LPS secretion still needs to be explored. Interestingly, *CHAC2* was first discovered to be the downstream for Adh-LPS-TLR4 pathways, indicating that the similar function of *CHAC2* may exist in other Gram-negative bacteria infection.

**Figure 6:**
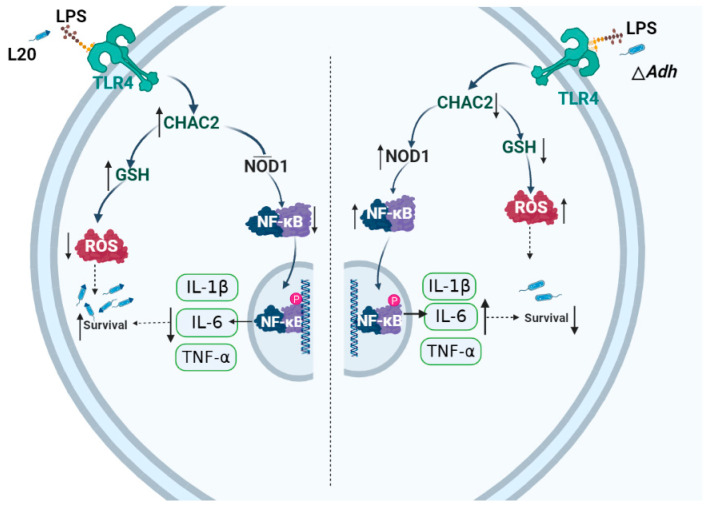
The mechanism of *A. pleuropneumoniae* immune escape mediated by *CHAC2*. The *Adh* domain of *A. pleuropneumoniae* increases *CHAC2* expression of macrophage through the LPS-TLR4 pathway. *CHAC2* can further suppress the ROS by GSH, and *IL-1β*, *TNF-α*, and *IL-6* expression via the NOD1/NF-κB signaling pathway in PAM. *CHAC2*, cation transport regulatory-like protein 2; GSH, Glutathione; ROS, reactive oxygen species. This figure was created with BioRender.com.

In summary, *Adh* of *A. pleuropneumoniae* induces the secretion of LPS binding to TLR4 to induce the *CHAC2* expression, which promotes GSH levels, and inhibits intracellular ROS to assists *A. pleuropneumoniae* survival in PAM, and also suppresses proinflammatory cytokines expression to attenuate inflammatory response through NOD1/NF-κB signaling pathway (Figure 6). This study clarified the mechanism by which *Adh* mediated the immune invasion, which lays a novel theoretical foundation for bacterial immunosuppressive effect, also provides a new target for prevention and treatment of *A. pleuropneumoniae*.

## Data Availability

Not applicable.

## Supplementary Materials

The following supporting information can be downloaded at: https://www.mdpi.com/article/10.3390/cells12050696/s1, Figure S1. QRT-PCR confirmation of the microarray analysis. (A) pcDNA-CHAC2 constructed by RT-PCR. (B) Identified by double digestion (C) and sequencing; Table S1. Primers used in this study; Table S2. ShRNA used in this study.
