# Supplementary material for: Adh Promotes Actinobacillus pleuropneumoniae Survival in Porcine Alveolar Macrophages by Inhibiting CHAC2-Mediated Respiratory Burst and Inflammatory Cytokine Expression
Source: Cells. 2023 Feb 22;12(5):696. doi: 10.3390/cells12050696 (PMC10001268; doi:10.3390/cells12050696)
Supplement: Supplementary file 1 [file cells-12-00696-s001.zip › Supplementary Files.pdf]

Supplementary Material

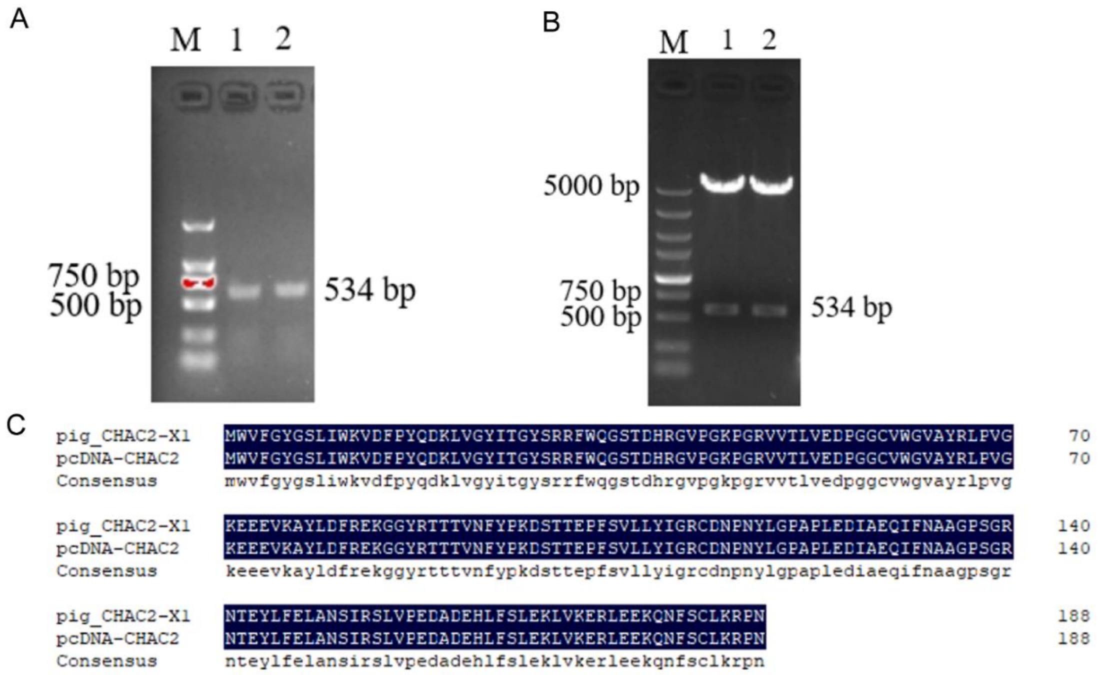

Fig S1. QRT-PCR confirmation of the microarray analysis. (A) pcDNA-CHAC2 constructed by RT-PCR. (B) Identified by double digestion (C) and sequencing.

Table S1. Primers used in this study

Primer name	Primer sequence (5' – 3')
CHAC2	F: TAGCGTTTAAACTT AAGCTT GCCACC ATGTGGGTTTTTGGTTATGGGTCCC
	R: GCTGGATATCTGCA GAATTC TCAATGATTAGGTCTTTTTAAGCAAC
CHAC2-mRNA	F: TTGGTCCTGCACCTCTGGAG
	R: TGCATCTTCTGGCACAAGGC
IL-6	F: GGAGACCTGCTTGATGAGAATC
	R: CAGCCTCGACATTTCCCTTAT
IL-1β	F: TCTGTACCTGTCTTGTGTGATG
	R: AAAGAGGGACATGGAGAAGC
TNF-α	F: CTACCTTGTTGCCTCCTCTTT
	R: GAGCAGAGGTTCAGTGATGTAG
IFN-γ	F: ACCTAAGAAAGCGGAAGAGAAG
	R: TAATTGTCATCCTGCCTGCA
IL-10	F: GGAGGTGAAGAGTGCCTTTAG
	R: AACTACATAGAAGCCTACATGACG
β-actin	F: CCACCCAGAAGACTGTGGAT
	R: AAGCAGGGATGATGTTCTGG

Table S2. ShRNA used in this study

Name	Target sequence
shRNA-CHAC2-A	GCTGGTCCGAGTGGAAGAAAT
shRNA-CHAC2-B	GCAAGCCTGGAAGAGTTGTGA
shRNA-CHAC2-C	GCCTGGAAGAGTTGTGACTCT
shRNA-control	CCTAAGGTTAAGTCGCCCTCG
